# The Mental Health and Wellbeing of Hazara Refugees in Australia: A Scoping Review

**DOI:** 10.1177/15248380251316905

**Published:** 2025-02-13

**Authors:** Grace Sultani, Milena Heinsch, Kate Vincent, Caragh Brosnan

**Affiliations:** 1 University of Newcastle, Callaghan, NSW, Australia; 2 University of Tasmania, Hobart, Australia

**Keywords:** mental health, wellbeing, trauma, Hazara, refugees, post-migration, Australia

## Abstract

Hazara refugees are highly vulnerable to trauma and other mental health challenges due to sustained ethnic and religious persecution. The post-migration difficulties experienced in Australia significantly heighten the risk of exacerbating these outcomes, while also highlighting the importance of identifying the various strengths and strategies that foster wellbeing. We conducted a scoping review to explore the mental health and wellbeing of Hazara refugees in Australia. A systematic database search was conducted across CINAHL Complete, MEDLINE, PsycINFO, Scopus, and Web of Science. Twenty-one articles met the eligibility criteria and were included in this review. Our findings draw attention to the distinct gendered mental health and wellbeing experiences of Hazara refugees. We highlight the emotional and psychological harm caused by visa insecurity and restrictions post-migration, as well as the detrimental impacts of racism and discrimination. We also discuss barriers to accessing support services, and identify key strategies used by Hazara refugees to promote their wellbeing in Australia. Lastly, findings highlight the collective experiences of suffering and growth experienced by Hazara refugees, and the cumulative impacts that all stages of forced migration may have on post-migration outcomes. In doing so, this review provides a critical overview of the mental health and wellbeing experiences of Hazara refugees in Australia and provides important recommendations for researchers and practitioners working with this population.

## Introduction

Refugees frequently experience profound trauma that places them at serious risk of developing mental health concerns. Prior to forced migration, refugees often face persecution, conflict, and violence (Lindert et al., 2016). In transit, many refugees are subjected to family separation, witness torture and killing, lose loved ones, and endure extreme environmental conditions (Li et al., 2016; Lindert et al., 2016). Post-migration, they can experience additional challenges, such as detention, deportation, delayed granting of refugee status, prolonged temporary protection status, and continued family separation (Li et al., 2016). These challenges occur alongside the pressures of second language acquisition, adapting to unfamiliar social and legal systems, seeking employment and financial stability, and experiencing discrimination (Li et al., 2016), constituting significant stressors that can profoundly impact overall wellbeing.

Despite the recognition that refugees can encounter hardship at every stage of their forced migration journey, research has historically focused predominantly on investigating the mental health and wellbeing impacts of pre-migration experiences (Hynie, 2018). However, recent years have seen an increasing exploration of post-migration experiences (Malm et al., 2020), revealing how post-migration environments frequently cause and exacerbate mental health and wellbeing concerns among refugees (Nowak et al., 2023). Importantly, research suggests that the post-migration experiences of different refugee populations can vary due to their distinct experiences of oppression. For example, ethnic or religious minority groups who have experienced discrimination, marginalization, and/or torture due to their ethnicity or religion, and who fled persecution connected to their minority status, have been found to exhibit higher levels of emotional and psychological distress post-migration than non-minority groups (Alemi et al., 2023; Berry & Taban, 2021).

Hazara refugees exemplify one minority group that is profoundly vulnerable to mental health and wellbeing concerns due to sustained ethnic and religious persecution (Zarak et al., 2020). Hazara people are an ethnic group from Afghanistan who are predominately Shia Muslim (Hakimi, 2022). The pervasive and systematic oppression of Hazara people in Afghanistan spans centuries, marked by frequent massacres, enslavement, and forced displacement; acts so severe that some researchers advocate for the recognition of genocide (e.g., Hakimi, 2022; Ibrahimi, 2017). The Taliban’s return to power in Afghanistan in 2021 heightened concerns for the safety of Hazara people, who now face escalated threats of violence and repression (Hakimi, 2023). These threats are causing increased rates of forced displacement among Hazara people (Hakimi, 2023), with many forced to seek safety in host or resettlement countries.

Australia has one of the largest communities of forcibly displaced Hazara people outside of Afghanistan, Iran, and Pakistan (Radford & Hetz, 2021). While the exact number of Hazara refugees in Australia is difficult to ascertain due to ongoing fears of ethnic persecution (Copolov et al., 2018; Spaaij et al., 2023), it is believed there are approximately 42,000 Hazara people currently residing in Australia (Australian Bureau of Statistics, 2021). Australia’s humanitarian program consists of offshore and onshore pathways for seeking refugee protection. The offshore pathway is designed for individuals whose refugee status is determined prior to arriving in Australia. The onshore pathway applies to those whose refugee status is determined after their arrival. The latter includes individuals who travel to Australia on a valid visa and apply for asylum, or who arrive in Australia without a valid visa and apply for asylum. While the migration experiences of offshore and onshore humanitarian applicants differ, both pathways present significant post-migration challenges.

The Australian Government has been widely criticized for its treatment of refugees, described by Macken (2020) as a “regime of cruelty and neglect that amounts to torture” (p. 9). Australia’s implementation and use of punitive policies, such as offshore processing, prolonged mandatory detention, temporary protection visas (TPVs), and barriers to family reunification highlight the structural violence and systematic hostility that refugees encounter (Barnes, 2022; Macken, 2020; Phillimore et al., 2023). In addition to the harm caused by these policies, refugees in Australia have often been subjected to negative, and at times oppressive, rhetoric in political and media discourse (Harding et al., 2025). This treatment, compounded by other post-migration challenges and the hardship experienced pre– and during migration, raises serious concerns about the mental health and wellbeing of Hazara refugees in Australia. It also highlights a critical need to recognize the various strengths and strategies drawn on by Hazara refugees post-migration to promote their wellbeing.

While research has begun to explore the mental health and wellbeing of Hazara refugees in Australia, to date, this literature has not been consolidated. Synthesis of this literature is critical to ensure that mental health research and practice in Australia can be adapted to respond to the key issues affecting the Hazara refugee community and to identify potential areas for future inquiry. To address this need, we used a scoping review methodology to explore the research question “What is known about the mental health and wellbeing of Hazara refugees in Australia?.”

## Methods

We considered a scoping review to be the most appropriate methodology for exploring the research question, given the need to determine the breadth and coverage of evidence in this field. Our review was guided by the frameworks of Arksey and O’Malley (2005), and Levac et al. (2010), and informed by the Preferred Reporting Items for Systematic Reviews and Meta-Analyses (PRISMA) extension for Scoping Reviews guidelines (Tricco et al., 2018). Throughout the review, we used the term “refugees” as an umbrella term for refugees, asylum seekers, and former refugees.

### Researcher Positionality

Before progressing, we would like to acknowledge our positionality as authors. We are White Australian or White Euro-Australian women who have grown up, and reside, in a Western culture. While not discussed in detail in this article, the first author has close personal ties to the Australian Hazara community. The remaining three authors are operating from an “outsider” perspective to this topic. Throughout the review process, we have tried to reflect on the ways in which our understanding of Hazara refugee experiences may have influenced the data synthesis, however, we recognize that there may still be blind spots in our reflexivity.

### Conceptualizing Mental Health and Wellbeing

The meanings and understandings ascribed to “mental health” and “wellbeing” vary significantly across cultures (e.g., Gopalkrishnan, 2018). Importantly, the ways in which these terms are conceptualized among refugee populations often differ from Western conceptualizations (Kerbage et al., 2020). For example, Kerbage et al. (2020) found that Syrian refugees described disruptions to mental health as a “normal” and inherent part of the refugee journey, rather than as symptomatic of mental ill-health, a perspective more prevalent in Western cultures. Similarly, among a sample of refugees from Afghanistan, Lavdas et al. (2023) found that participants did not view mental health concerns through a medical lens, but rather, saw these concerns as challenges that could be improved through informal social and emotional support. Mental health researchers are, therefore, cautioned against the excessive pathologizing of refugee suffering (Papadopoulos, 2007) and encouraged to carefully consider how mental health is conceptualized by the population they are engaging with.

In refugee studies, mental health is often operationalized in relation to wellbeing (Siddiq et al., 2023), suggesting that it is not always possible, or effective, to separate the two terms. While we recognize the distinctions between mental health and wellbeing (Keller, 2020), this review uses these terms together to facilitate a more comprehensive account of the wide-ranging experiences of Hazara refugees in Australia.

### Identifying Relevant Studies

A systematic search was conducted in May 2023 using the electronic databases of CINAHL Complete, MEDLINE, PsycINFO, Scopus, and Web of Science. The search strategy was established prior to undertaking the search, and included the terms: (“wellbeing” or “well being” or “well-being” or “mental health” or “mental illness” or “mental health issue*” or “mental health disorder*” or “mental health problem*” or “emotional health” or “stress” or “anxiety” or “depression” or “post-traumatic stress disorder*” or “PTSD” or “distress” or “psychological distress” or “emotional distress” or “trauma” or “resilience” or “transformation” or “strength” or “coping” or “identity” or “belonging” or “post-traumatic growth” or “posttraumatic growth” or “PTG” or “positive change” or “benefit finding” or “stress-related growth” or “adversarial growth” or “positive psychological change*” or “perceived benefits” or “transformational coping”) AND (“Hazara” or “Hazaras” or “Hazara people” or “Afghan” or “Hazara-Afghan” or Afghan-Hazara”) AND (“refugees” or “refugee*” or “asylum seeker*” or “humanitarian entrant*” or “displaced person” or “displaced people” or “forcibly displaced person” or “forced migrant” or “human migration” or “former refugee” or “stateless person” or “migrant*” or “migrat*” or “post-migration” or “displace*” or “resettlement” or “resettle*” or “journey” or “movement”).

The search and screening process was repeated in January 2024 to update the findings.

### Article Selection

We initially intended to include both Australian and international literature in this review. Articles were eligible if they: (a) were published in English, (b) were peer-reviewed empirical studies (qualitative, quantitative, or mixed methods), (c) were dissertations or theses, (d) were review papers, (e) were book chapters, (f) included Hazara refugees, asylum seekers, or former refugees of all ages, and (g) explored the mental health and wellbeing of Hazara refugees post-migration. All study designs were included in this review in line with our aim to provide a comprehensive overview of the available literature. Studies were excluded if they: (a) were published in a language other than English, (b) were not reporting on post-migration experiences, (c) were abstracts only, (d) included non-Hazara participants, (e) included participants of a non-refugee background, and (f) did not specify the ethnicity of participants as Hazara. No limitation was placed on the publication date.

After de-duplication, the records were uploaded to Covidence (Veritas Health Innovation Ltd., Melbourne, Victoria, Australia) for title and abstract screening by two authors. Potentially eligible articles underwent independent assessment for eligibility using the selection criteria. Discrepancies in decision-making were resolved through reviewer discussion with the senior author. The reference lists of all eligible articles were manually screened to identify articles missing in the database search.

Upon conducting the initial search and screening, we found that all except two studies meeting our eligibility criteria were conducted in Australia. To better contextualize the findings to the Australian refugee context, we decided to limit inclusion to Australian literature. Consequently, two international studies (Chiovenda, 2021; Kassam & Nanji, 2006) were excluded.

### Data Charting and Synthesis

Data were extracted using a purpose-designed spreadsheet. Two authors extracted the following study characteristics: (a) author and date, (b) aim of article, (c) research design and methods, (d) sample size, gender, and age, (e) time in Australia, and (f) religion. Findings were analyzed using thematic synthesis, which consisted of three overlapping stages (Thomas & Harden, 2008). Guided by the approach proposed by Thomas and Harden (2008), the findings of the included studies were first coded line-by-line according to their meaning and content. The codes were then grouped into categories based on their similarities and differences, resulting in the development of descriptive themes. Finally, analytical themes were generated to provide insights that extended beyond the original content of the studies. The analytical themes were developed through the grouping of descriptive themes and were finalized through discussion among the reviewers.

## Results

### Search Results

**Figure 1.**
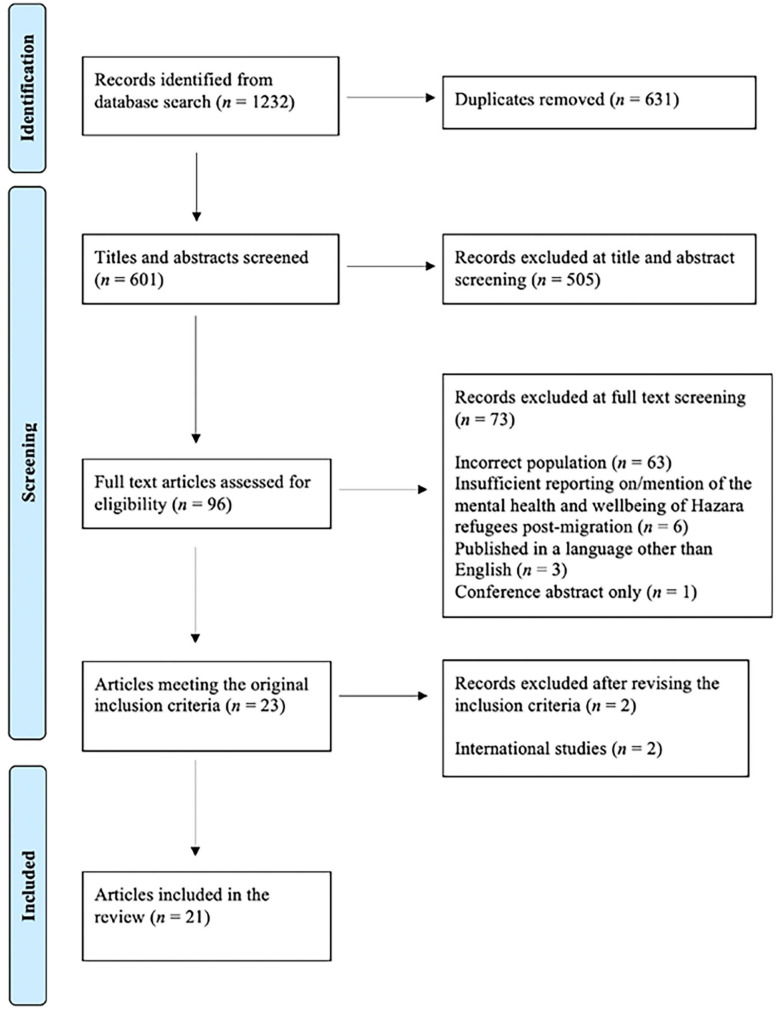
PRISMA flowchart. *Note.* PRISMA = Preferred Reporting Items for Systematic Reviews and Meta-Analyses.

The PRISMA flowchart (Figure 1) details the process of study inclusion and exclusion. The electronic database searches resulted in 1,232 potentially eligible studies; 631 following de-duplication. A further 505 studies were excluded during the title and abstract screening. Of the remaining 96 studies, 73 were excluded during the full-text screening for the following reasons: (a) incorrect population (*n* = 63), (b) insufficient reporting on/mention of the mental health and wellbeing of Hazara refugees post-migration (*n* = 6), (c) published in a language other than English (*n* = 3), and (d) conference abstract only (*n* = 1). In total, 23 articles met the eligibility criteria. As noted above, a decision was later made to limit the inclusion criteria to Australian literature, resulting in the exclusion of two international studies. Thus, a total of 21 articles were included in this review.

Three sets of articles reported on the same studies (study 1: Copolov & Knowles, 2021, 2023; study 2: Hamrah et al., 2021a and Hamrah et al., 2021b; study 3: Phillips, 2005, 2011, 2019). All were included in the review.

### Study Characteristics

As shown in Table 1, studies included in this review used either qualitative (*n* = 18) or quantitative (*n* = 3) approaches. No mixed-methods studies were identified for inclusion. Most studies used a cross-sectional design (*n* = 17). Qualitative studies used interviews (*n* = 13), interviews and focus groups (*n* = 3), and interviews and participant observation (*n* = 2). Quantitative studies used surveys (*n* = 3).

**Table 1.** Summary of the Key Characteristics of Included Articles.

Author and Date	Aim of Article	Research Design and Methods	Sample Size, Gender, and Age	Time in Australia	Religion
Burford-Rice et al. (2022)	To examine existing formal and informal help-seeking patterns among refugee women from Afghanistan, to explore how they conceptualize mental health issues, and whether this influences help-seeking behaviors.	Qualitative semi-structured interviews.	11 participants 100% female Ages 18–60	5 months–16 years	Not stated
Copolov et al. (2018)	To determine the predictors and mediators of personal wellbeing for young Hazara refugees in Australia.	Cross-sectional survey (using Personal Wellbeing Index-Adult (PWI-A), Acculturation and Resilience Scale (AARS), and Reactions of Adolescents to Traumatic Stress Questionnaire (RATS)).	70 participants 71% male Ages 16–30	1–15 years	94% Shia Muslim, 3% Sunni Muslim 3% no religious affiliation.
Copolov and Knowles (2021)	To explore the resettlement experiences and adaptation of young Hazara refugees.	Qualitative semi-structured interviews.	18 participants 50% female Ages 18–30	1–16 years	Shia Muslim
Copolov and Knowles (2023)	To explore psychological distress among young Hazara refugees.	Qualitative semi-structured interviews.	18 participants 50% female Ages 18–30	1–16 years	Shia Muslim
Goodall and Hekmat (2021)	To explore the life histories of Hazara women in Australia, tracing the environmental dimensions of their refugee journeys.	Qualitative in-depth oral history interviews.	12 participants 100% female Ages 16–56	Full range is not stated.	Not stated
Hamrah et al. (2021a)	To examine the occurrence and correlates of depressive symptoms among refugees from Afghanistan resettled in Australia.	Cross-sectional survey (using Hopkins Symptom Checklist (HSCL-25), and Post-Migration Living Difficulties Scale (PMLD))	66 participants 74% male Ages 18 to >50	1–5 years (for 97% of participants). Full range is not stated.	Not stated
Hamrah et al. (2021b)	To examine the occurrence and correlates of post-traumatic stress disorder among refugees from Afghanistan resettled in Australia.	Cross-sectional survey (using Post-Migration Living Difficulties Scale (PMLD), and Impact of Event Scale-Revised (IES-R))	66 participants 74% male Ages 18 to >50	1–5 years (for 97% of participants). Full range is not stated.	Not stated
Iqbal et al. (2012)	To explore the resettlement experiences of female Hazara adolescents.	Qualitative in-depth semi-structured interviews (*n* = 8 participants) and focus groups (*n* = 8 participants).	14 participants 100% female Ages 14–17	5 years or less	Not stated
Mackenzie and Guntarik (2015)	To explore Hazara refugees’ experiences of transition in Australia.	Qualitative interviews.	4 participants^a^ 100% male Age range is not stated.	10–20 years	Not stated
Neve (2022)	To investigate the challenges and strategies of young asylum seekers from Afghanistan during their resettlement.	Qualitative semi-structured interviews and participant observation.	3 participants^b^ 100% male Ages 20–21	Full range is not stated.	Not stated
Parkes (2020)	To examine the lived experiences of Hazara refugees in relation to their migration, relocation, and individuation in Australia.	Ethnography, structured and semi-structured interviews.	3 participants 100% male Age range is not stated.	Full range is not stated.	Shia Muslim
Phillips (2005)	To explore the narrative of a Hazara refugee in Australia.	Qualitative oral history interview.	1 participant male Age 21	Full range is not stated.	Shia Muslim
Phillips (2011)	To explore oral histories of Hazara refugees in Australia.	Qualitative oral history interviews.	3 participants 100% male Age range is not stated.	Full range is not stated.	Shia Muslim
Phillips (2019)	To explore experiences of grief with a Hazara refugee.	Qualitative oral history interview.	1 participant Male Age range is not stated.	Full range is not stated.	Shia Muslim
Radford and Hetz (2021)	To explore how Hazara refugees negotiate multiple identities and belonging in Australia.	Qualitative semi-structured interviews.	20 participants Gender breakdown is not stated. Age range is not stated.	Full range is not stated.	Muslim (not specified), and no religious affiliation.
Russo et al. (2015)	To explore the emotional wellbeing and support needs of refugee women from Afghanistan living in Australia throughout their pregnancy, birth, and early motherhood.	Qualitative focus groups (*n* = 28 participants) and in-depth interviews (*n* = 10 participants).	38 participants 100% female Ages 20–38	1–6 years	Not stated
Saberi et al. (2021)	To explore the understanding of mental health and barriers to accessing primary mental health care among young Hazara refugees in Australia.	Qualitative semi-structured interviews (*n* = 2 participants) and focus groups (*n* = 15 participants).	17 participants 52% male Ages 18–29	Full range is not stated.	Not stated
Saniotis and Sobhanian (2008)	To locate storytelling in the lives of refugee women from Afghanistan living in Australia.	Qualitative interviews.	10–12 participants^c^ 100% women Ages 26–42	6 months–1.5 years	Not stated
Shahimi et al. (2026)	To explore how young adult Hazara refugees view their identity and resilience during forced migration, and how their environment may have influenced these perceptions.	Qualitative in-depth semi-structured interviews.	15 participants 53% male Ages 20–28	6–18 years	Shia Muslim
Spaaij et al. (2023)	To examine how Hazara refugee men’s involvement in ethno-specific informal sport influences their post-migration experiences of (non)belonging in Australia.	Qualitative fieldwork observations (*n* = 10–50 participants present) and semi-structured interviews (*n* = 11 participants).	100% male Ages 25–70	3 months–30 years	Not stated
Wilson et al. (2023)	To explore understandings and applications of self-compassion and self-coldness among Hazara refugees in Australia.	Qualitative semi-structured interviews.	11 participants 82% male Age range is not stated.	Full range is not stated.	91% Shia Muslim, and 9% no religious affiliation.

a Article is based on a wider study with 30 participants.

b Article is based on a wider study with 41 participants.

c Authors state “10 to 12 women were interviewed”, p. 2.

Across the included studies, sample sizes ranged from 1 to 70. Participant ages ranged from 14 to 70 years, and time in Australia ranged from 3 months to 30 years. Seven studies included only men, five included only women, and eight studies included both men and women. One study did not state the gender breakdown of participants. Ten studies documented the religion of participants. Ten studies provided a detailed discussion of the way gender may have impacted and shaped mental health and wellbeing experiences. Six studies provided a detailed examination of how past trauma exposure, such as pre-migration and transit experiences, impacted mental health and wellbeing post-migration. See Supplementary Material for a summary of key findings from each article, relating to the review.

Thematic synthesis of the included studies resulted in six interconnected themes: (1) gendered mental health and wellbeing experiences—“I have more responsibilities”; (2) impact of visa insecurity and restrictions— “I still worry about my future here”; (3) racism and discrimination post-migration—“I honestly don’t feel safe sometimes”; (4) barriers to accessing support services—“They just make me sad”; (5) strategies to promote wellbeing post-migration—“That’s where I literally forget my old pain”; and (6) collective Hazara suffering and strength—“Our blood has been shed because we are Hazaras.” The themes are presented here in narrative form and anchored with quotes to foreground the voices of participants in the included articles. Due to the limited number of quantitative studies included in this review, qualitative and quantitative findings are presented together.

#### Gendered Mental Health and Wellbeing Experiences: “I Have More Responsibilities.”

Synthesis of the included studies drew attention to the gendered mental health and wellbeing experiences of Hazara refugees in Australia, with these experiences being particularly impacted by expected gender roles. Participants described how prevailing gender roles for men continued post-migration, with an expectation that they would financially provide for their families (Copolov & Knowles, 2021; Wilson et al., 2023). They described this expectation as highly stressful: “I have more responsibilities . . .to help support my family back in Afghanistan and to cope with my life here. . . “send me money, send me money, send me money.” I need the money here as well” (Copolov & Knowles, 2023, p. 117). The financial pressure for men to provide for their families, described by some participants as “self-sacrificing” (Wilson et al., 2023), also narrowed occupational and educational choices in Australia, leading to a lack of fulfillment: “I just got a job in a meat factory and that was quick and that’s not what I was looking for at the moment. . .I want a job which I can enjoy” (Copolov & Knowles, 2021, p. 192). While not reported among women, some men engaged in substance use, including alcohol, smoking, and illicit drug use to minimize feelings of psychological distress (Copolov & Knowles, 2023).

Women had mixed experiences relating to their expected gender roles in Australia. For some, gender roles expanded to include educational and employment opportunities, often leading to a sense of pressure to succeed (Copolov & Knowles, 2021; Iqbal et al., 2012; Mackenzie & Guntarik, 2015): “I always felt this form of anxiousness that I am not doing enough. . . I have a lot of opportunities here” (Copolov & Knowles, 2023, p. 117). For others, the expectation from family members to perform domestic duties impacted opportunities to pursue education: “Some girls want to receive education, but parents think that you should have limited education. . .learn household skills and go and marry a boy and have kids” (Iqbal et al., 2012, p. 5). The latter was attributed by participants to a cultural pressure in the Hazara community, and often led to feelings of frustration and helplessness (Copolov & Knowles, 2023; Iqbal et al., 2012). Only one of the three quantitative studies identified significant gender differences in relation to mental health and wellbeing outcomes. Hamrah et al. (2021a) found that high-level depressive symptoms were significantly more common among women (41.2%) than men (14.3%).

#### Impact of Visa Insecurity and Restrictions: “I Still Worry About My Future Here.”

Many studies described the detrimental impacts of the insecurity and restrictions caused by temporary protection status on mental health and wellbeing. Participants reported that living with TPVs resulted in a constant fear of deportation, and kept them intimately connected to painful memories of victimization, due to the continuous need to recount and prove their experiences of persecution when applying for subsequent visas (Phillips, 2011). Among participants, TPVs hindered employment prospects post-migration (Shahimi et al., 2026): “Looking for a job they always ask me ‘what kind of visa do you have?’” (Neve, 2022, p. 461). They also negatively impacted opportunities for education: “My visa makes study expensive. . .so I cannot” (Neve, 2022, p. 461). As a result, TPVs led to feelings of ongoing uncertainty and insecurity post-migration (Neve, 2022; Wilson et al., 2023): “I still worry about my future here. . .I can’t make any plans” (Phillips, 2005, p. 31). They also contributed to feelings of exclusion and isolation (Phillips, 2019). It is important to note that while permanent protection or citizenship did provide a sense of security and freedom for some Hazara refugees (Phillips, 2019), others continued to experience feelings of instability and anxiety post-migration (Goodall & Hekmat, 2021).

While holding TPVs, participants were also barred from sponsoring their families to come to Australia (Copolov & Knowles, 2021). The distress caused by this restriction was compounded by participants’ grave concerns for the safety of family members overseas, and their inability to leave Australia to reunite due to their temporary protection status (Copolov & Knowles, 2021; Phillips, 2005, 2011; Saberi et al., 2021). As a result, many participants experienced long, painful periods of separation from their families, with some not knowing if or when they would ever reunite: “How can I see my mother again? It should be possible to see her again. I don’t have nothing else; I don’t have any more of my life, just once can I see my mother?” (Phillips, 2011, p. 195). In their quantitative study, Hamrah et al. (2021b) found that family separation was strongly associated with post-traumatic stress disorder.

#### Racism and Discrimination Post-Migration: “I Honestly Don’t Feel Safe Sometimes.”

Across the included studies, Hazara refugees shared their experiences of racism and discrimination in Australia. Religious discrimination was noted as having profound negative impacts on the sense of security among Hazara refugees, affecting their ability to safely access certain spaces, such as workplaces, schools and universities, and community gatherings (Iqbal et al., 2021; Shahimi et al., 2026; Spaaij et al., 2023): “I honestly don’t feel safe sometimes” (Copolov & Knowles, 2021, p. 190). In particular, the negative stereotypes and media discourse portraying Muslim people as “terrorists” often resulted in Hazara refugees feeling intimidated, threatened, excluded, and unsafe in Australia, ultimately affecting their sense of belonging (Copolov & Knowles, 2021; Shahimi et al., 2026). Hazara women spoke of experiencing racist stereotyping based on their gender and cultural/national identity: “It was a psychology class, and the teacher would come up . . . oh why you even studying so hard? All Afghans girls at the end of their day they would get married, have kids, make a family” (Shahimi et al., 2026, p. 12). Hazara men also described experiences of racist stereotyping in the community, including at places of education and work (Shahimi et al., 2026; Spaaij et al., 2023). While encounters of racism and discrimination affected both women and men, some participants noted that women wearing headscarves were particularly likely to face discrimination in Australia as their Muslim identity was more visibly recognizable: “For women especially because we are wearing the whole outfit with our headscarves compared to the men, they are just a normal typical guy going out” (Copolov & Knowles, 2021, p. 190). Despite the devastating impacts of racism and discrimination, some Hazara refugees did express a sense of belonging and acceptance within the Australian community. They explained that the discrimination they faced post-migration, while still devastating, was less dangerous *in comparison* to the discrimination they suffered in Afghanistan and neighboring countries, which could involve being “pulled off a bus and shot by Sunni extremists” (Radford & Hetz, 2021, p. 386).

#### Barriers to Accessing Support Services: “They Just Make Me Sad.”

Hazara refugees discussed various barriers to accessing support services in Australia. Hamrah et al. (2021b)found that while most participants with a probable diagnosis of post-traumatic stress disorder recognized that they were experiencing mental health difficulties, less than half (46.9%) sought professional help. Participants raised concerns that their post-migration needs in Australia were often overlooked by mental health professionals due to a heavy or sole focus on past experiences: “She [the psychologist] asks a lot of questions about my family and my past but I don’t have any problem with my family” (Copolov & Knowles, 2023, p. 120). As a result, some participants felt that their mental health concerns were not adequately understood or addressed, which exacerbated distress symptoms, and reduced trust in health care services: “They just make me sad” (Copolov & Knowles, 2023, p. 119). Barriers to accessing support services were influenced by a concern that disclosures of mental ill-health may affect an individual’s refugee status in Australia: “People are scared they might be deported so they keep everything to themselves” (Copolov & Knowles, 2023, p. 118). Accessing support was also influenced by a mental health stigma in the Hazara culture (Copolov & Knowles, 2021; Russo et al., 2015; Saberi et al., 2021), expressed through terminology such as “crazy” and “mad”: “If they go to any service for help, other people will think wrong about her and her reputation will be very bad in my community” (Burford-Rice et al., 2022, pp. 84–85). Many participants described their mental and physical health as interconnected and interdependent, often referring to physical symptoms in the brain or heart to describe psychological distress (Burford-Rice et al., 2022; Copolov & Knowles, 2023): “My head is heavy. . .I feel that my brain is not working” (Saniotis & Sobhanian, 2008, p. 4). Due to these physical manifestations of psychological distress, some participants chose to seek and receive medical treatment, rather than mental health support (Burford-Rice et al., 2022). Disclosures of family violence were also impacted by stigma and cultural expectations that discouraged discussing such issues: “If they are facing domestic violence, they will keep with themselves. . . she would never raise her voice because she has grown up in that environment. . . if she has raised her voice then people will say something about her.” (Burford-Rice et al., 2022, p. 86).

#### Strategies to Promote Wellbeing Post-Migration: “That’s Where I Literally Forget My Old Pain.”

Hazara refugees described the ways in which they coped with psychological distress and promoted their wellbeing post-migration, through religion (Burford-Rice et al., 2022; Copolov & Knowles, 2023; Parkes, 2020; Russo et al., 2015), social support (Copolov & Knowles, 2021, 2023; Neve, 2022; Russo et al., 2015), and sport (Copolov & Knowles, 2023; Parkes, 2020; Shahimi et al., 2026; Spaaij et al., 2023). First, religious practices such as prayer or reading a religious text created a sense of calm and hope (Burford-Rice et al., 2022; Copolov & Knowles, 2023; Russo et al., 2015). It also provided participants with a source of guidance (Parkes, 2020; Russo et al., 2015): “If you turn to God he will always answer your questions” (Burford-Rice et al., 2022, p. 88). Second, many participants highlighted social engagement as their preferred avenue for receiving emotional support in Australia, explaining how social support from friends and family, particularly within the Hazara community, helped them to cope with hardship by facilitating a sense of connection and belonging (Russo et al., 2015; Spaaij et al., 2023): “When we are sitting together, sometimes we don’t need to talk about everything, when I say the beginning of my thoughts, he can read the end of it” (Shahimi et al., 2026, p. 13). Quantitative results supported these findings by revealing how depressive symptoms were significantly increased by isolation (defined as being or feeling alone) (Hamrah et al., 2021a). Social support also provided participants with a source of practical assistance in Australia, such as help in locating employment and housing (Neve, 2022). Lastly, several studies identified the role of sport in improving Hazara refugees’ mental health and wellbeing post-migration, acting as a coping mechanism that provided a safe space and expanded their social networks (Copolov & Knowles, 2023; Parkes, 2020; Spaaij et al., 2023): “[Football] was the language I could communicate with people. . .that’s where I literally forget my old pain” (Shahimi et al., 2026, p. 13). Sport also created a sense of purpose among some participants and helped them develop a new sense of identity in Australia (Parkes, 2020).

#### Collective Hazara Suffering and Strength: “Our Blood Has Been Shed Because We Are Hazaras.”

Hazara refugees described a sense of collective suffering and strength within their community. Participants felt bound by their shared memories of persecution, and often associated these memories with their mental health and wellbeing post-migration (Phillips, 2011): “The terror attacks are affecting us, it is not new, it’s from thousands of years. . .total injustice, so that causes depression. . .if a Hazara is killed” (Saberi et al., 2021, p. 452). They often positioned their own experiences of distress within the context of the broader Hazara community, and memories of persecution against the community were described as having an ongoing and detrimental impact on their mental health and wellbeing (Phillips, 2011, 2019; Saberi et al., 2021): “We watched bodies of our own people being torn to pieces and we saw that as kids. . .It’s so traumatic. . .nothing I can do can erase those memories” (Shahimi et al., 2026, p. 6). These shared experiences of persecution led many participants to experience a strong connection with their Hazara identity in Australia (Phillips, 2011; Radford & Hetz, 2021; Mackenzie & Guntarik, 2015): “Our blood has been shed because we are Hazaras” (Copolov & Knowles, 2021, p. 193). This was often accompanied by a preference for identifying as “Hazara” rather than using the terms “Afghan” or “Afghanistan,” which some participants understood to mean “Pashtun” (the largest ethnic group of Afghanistan) and “Pashtun land” (“land of their oppressors”) (Radford & Hetz, 2021, p. 382).

Participants noted that their identity as Hazara refugees was simultaneously associated with both suffering, pain and discomfort, and resilience, determination and courage (Shahimi et al., 2026). Hazara refugees described how the hardship they had endured provided them with a source of motivation and determination to overcome post-migration challenges in Australia (Neve, 2022): “It provided that fuel that I need. . .To have goals, to strive to struggle. . .It’s through my own traumatic personal experience” (Shahimi et al., 2026, p. 7). For many, intergenerational trauma and past experiences of persecution also shaped career choices in Australia, with participants seeking to “give back” to the Hazara community (Mackenzie & Guntarik, 2015; Shahimi et al., 2026). For example, Hazara refugees engaged in advocacy and political efforts to raise awareness of the issues affecting the Hazara community (Mackenzie & Guntarik, 2015): “I can help. . .That connected me to [the community]. . .made me feel that I am making a difference” (Shahimi et al., 2026, p. 14). However, it is important to note that despite a desire to raise awareness about issues affecting the Hazara community, some individuals felt silenced by fear of jeopardizing their visa status or endangering the safety of their family overseas: “If I say something according to the politics and circumstances, I’m scared of my own life, not just because of me . . . but because of my family who is still living in Afghanistan” (Phillips, 2011, p. 195).

## Discussion

This review synthesized insights from 21 articles, with findings drawing attention to the gendered nuances of mental health and wellbeing among Hazara refugees, and the emotional and psychological harm caused by visa insecurity and restrictions post-migration. Findings also highlight the pervasive effects of racism and discrimination, identify barriers to accessing support services, and discuss the various strategies Hazara refugees draw on to promote their wellbeing post-migration. Furthermore, this review highlights the interconnected experiences of suffering and growth among Hazara refugees in Australia. A summary of critical findings is presented in Table 2, followed by implications for research, practice, and policy in Table 3.

**Table 2.** Critical Findings.

Focus	Critical Findings
Past trauma	The mental health and wellbeing of Hazara refugees were significantly impacted by their past experiences of trauma, including during pre-migration and transit to Australia.
Visa insecurity and restrictions	Findings highlight the emotional and psychological harm caused by visa insecurity and restrictions, including a constant fear of deportation, long periods of family separation, the inability to propose the resettlement of family to Australia, and difficulties accessing education and employment.
Gender	Hazara refugee women and men had distinct mental health and wellbeing experiences, often influenced by prevailing gender roles. For example, many Hazara men experienced financial pressure to provide for their families, and some women were expected to perform domestic duties that impacted opportunities to pursue education.
Racism and discrimination	Experiences of racism and discrimination had detrimental impacts on the mental health and wellbeing of Hazara refugees in Australia, leading to feelings of exclusion and non-belonging, and affecting their ability to safely access the community.
Collective suffering and strength	Findings highlight how suffering and strength were experienced collectively among many Hazara refugees as a result of being connected by their shared memories of persecution.
Co-existing vulnerabilities and strengths	Hazara refugees’ experiences of suffering and growth interacted with, and occurred alongside, one another.

**Table 3.** Implications for Research, Practice, and Policy.

Focus	Implications for Research, Practice, and Policy
Past trauma	Researchers and practitioners must recognize the continuum of the refugee experience, and the cumulative impacts different stages of forced migration may have on post-migration mental health and wellbeing outcomes.
Visa insecurity and restrictions	There is a critical need for policy reform to provide equal opportunities for family reunification among *all* refugee populations. Additionally, there is also a need for researchers and practitioners to not only consider the mental health and wellbeing impacts of refugees’ current immigration or visa status but to also consider the continuing systemic harm and penalization they may face due to their mode and time of arrival in Australia.
Gender	Researchers are encouraged to use a gender-sensitive approach that thoroughly examines the impact of gendered socio-cultural challenges and strengths on mental health and wellbeing outcomes. A gender-sensitive approach may involve providing equal opportunities for participation among women and men, achieving an adequate representation of both groups in the participant sample, examining gender differences through data collection and analysis, and using gender-sensitive language.
Racism and discrimination	Urgent policy and community action are needed to address racism and discrimination in the post-migration environment. This may include developing community-level anti-discrimination initiatives in the Australian community, establishing community engagement and education initiatives that focus on building relationships between refugees and the wider Australian community, ensuring adequate funding for appropriate counseling and support for refugees experiencing discrimination, and ceasing discriminatory language toward refugees in political and media discourse.
Collective suffering and strength	Researchers and practitioners are urged to draw on a sociocultural and historical framework that encourages insight into the shared, complex, and intergenerational traumas experienced by Hazara refugees. This framework may also provide an opportunity to identify and draw on community strengths, resources, and pathways to recovery.
Co-existing vulnerabilities and strengths	Concepts such as “post-traumatic growth,” or similar, may be useful in extending current understandings of the complex ways in which Hazara refugees experience positive psychological changes post-migration.

A critical finding of this review was that the mental health and wellbeing of Hazara refugees’ post-migration was shaped by past trauma experienced pre-migration and during transit. For example, participants often associated experiences and memories of persecution pre- and during migration with their distress in Australia (Phillips, 2011; Saberi et al., 2021). It is noteworthy that only 6 of the 21 articles included in this review provided a detailed examination of the cumulative impact of Hazara refugees’ past experiences on their mental health and wellbeing post-migration. The broader literature also reflects a limited focus on the cumulative trauma experienced by other refugee populations, with Saadi et al. (2021) calling for recognition of the interconnected experiences of pre-migration trauma, trauma experienced during transit, and post-migration trauma. Given the sustained persecution and oppression faced by Hazara refugees, it is imperative that researchers and practitioners recognize the continuum of the refugee experience, including the cumulative impacts that all stages of forced migration may have on post-migration outcomes.

This review highlighted the emotional and psychological harm caused by visa insecurity and restrictions (Copolov & Knowles, 2021; Neve, 2022; Phillips, 2005, 2011; Shahimi et al., 2026), aligning with findings from the broader refugee literature (e.g., Kenny et al., 2022). While one of the included studies noted that participants on TPVs were unable to sponsor their families to come to Australia (Copolov & Knowles, 2021), none of the studies explored the long-term systemic barriers to family reunification that may persist for some Hazara refugees even after they receive permanent residency or citizenship. For example, through Australia’s Special Humanitarian Program, which is the primary pathway through which refugees can apply for family reunification, individuals who arrived by boat on or after August 13, 2012, are *permanently* barred from proposing the resettlement of family members to Australia (Wickes et al., 2019). In doing so, this policy disadvantages one of the most vulnerable refugee groups in Australia (Wickes et al., 2019) and creates ongoing distress. Given the profound positive impact of family reunification on the mental health and wellbeing of refugees (Phillimore et al., 2023), there is a critical need for policy reform to ensure equal opportunities for family reunification across *all* refugee populations. This discussion also highlights the need for researchers and practitioners to consider not only the mental health and wellbeing impacts of refugees’ current immigration or visa status but also the continuing systemic harm and penalization they may face due to their mode and time of arrival in Australia.

Findings from this review emphasized how the mental health and wellbeing of Hazara refugees were often influenced by prevailing gendered roles and expectations. Nevertheless, only 10 of the included articles provided a detailed discussion of the way participants’ gender may have impacted or shaped their mental health and wellbeing outcomes in Australia. The broader literature highlights a similar trend where research undertaken with refugee populations is often “gender-blind,” thereby obscuring the nuanced and complex differences in the post-migration experiences of women and men (Cheung & Phillimore, 2017). This gap is particularly concerning since gender shapes all phases of the refugee journey (Pittaway & Bartolomei, 2018). This review, guided by the recommendation of Al-Krenawi and Bell (2023), highlights the importance of gender-sensitive research to ensure thorough examination of the impact of gendered socio-cultural challenges and strengths on the mental health and wellbeing of Hazara refugees. In research that includes women and men, a gender-sensitive approach may involve: (a) providing equal opportunities for participation among both groups; (b) achieving adequate representation of women and men in the sample; (c) examining gender differences through data collection and analysis; and (d) using gender-sensitive language (Heidari et al., 2016).

This review shed light on the detrimental impacts of racism and discrimination on the mental health and wellbeing of Hazara refugees in Australia. These experiences led to feelings of exclusion and non-belonging and affected some refugees’ ability to safely access the community (Copolov & Knowles, 2021; Iqbal et al., 2012; Shahimi et al., 2026; Spaaij et al., 2023). The harmful effects of racism and discrimination have also been identified in the wider Australian literature, where Ziersch et al. (2020) found that 90% of refugees who had faced discrimination associated this experience with worsened physical or mental health outcomes. They described discrimination as a pressing post-migration health concern requiring immediate action (Ziersch et al., 2020). Such action may include, but is not limited to: (a) developing community-level anti-discrimination initiatives in the Australian community; (b) establishing community engagement and education initiatives that focus on building relationships between refugees and the wider Australian community; and (c) ensuring adequate funding for appropriate counseling and support for refugees experiencing discrimination (Ziersch et al., 2020). With Australia’s government policies and negative political/media discourse fostering an environment where discrimination can flourish, it is also imperative that discriminatory and inflammatory language toward refugees is immediately ceased (Ziersch et al., 2020). These measures, which should be further developed in collaboration with refugee communities (Ziersch et al., 2020), are imperative for safeguarding the mental health and wellbeing of refugee populations, such as Hazara people, from further harm within Australia.

A key finding of this review was the ways in which suffering and strength were experienced collectively among Hazara refugees, extending beyond an individual level. For example, participants felt connected by their shared experiences and often positioned their own distress within the context of the broader Hazara community (Phillips, 2011, 2019; Saberi et al., 2021). Experiences of collective suffering have also been documented in the wider refugee literature, with researchers warning that examining trauma (a) solely on an individual level, and (b) related to a sole isolated event, as predominant through a Western lens, is not sufficient in understanding the mental health and wellbeing of refugees post-migration (Theisen-Womersley, 2021). When working alongside groups who have experienced ongoing systematic violence based on their racial or ethnic identity, such as Hazara refugees, researchers and practitioners are urged to draw on a sociocultural and historical framework (Ortega-Williams et al., 2021). This approach encourages insight into the shared “cumulative, complex, and persistent” traumas that may span multiple generations (Ortega-Williams et al., 2021, p. 7), while simultaneously providing an opportunity to identify and draw on community resources and pathways to recovery.

Furthermore, findings from this review shed light on the co-existing nature of the vulnerabilities and strengths of Hazara refugees. Participants of the included studies highlighted how their experiences of suffering and growth interacted with, and occurred alongside, one another. This co-occurrence has been commonly reported among refugee populations (e.g., Maung et al., 2021; Sultani et al., 2024). These findings emphasize the danger of researchers or practitioners focusing solely on the vulnerabilities of refugees, and thus overlooking the agency, strength, and capacity for growth that exists within refugee communities (Papadopoulos, 2007; Pittaway & Bartolomei, 2018; Theisen-Womersley, 2021). Similarly, focusing only on experiences of strength and growth without recognition of the profound suffering experienced by refugees risks the over-simplification of their experiences. Concepts such as “post-traumatic growth” (Tedeschi & Calhoun, 1995) or similar, that recognize the occurrence of growth in the context of suffering and struggling (Tedeschi et al., 2018), may be useful in extending current understandings of the complex ways in which Hazara refugees experience positive psychological changes post-migration.

Lastly, while several reviews have examined the mental health and wellbeing of broader refugee populations, including those with Hazara participants within larger cohorts, the current review undertook a more focused approach to specifically examine the experiences of Hazara people. The wider refugee literature reports findings that align with some of those discussed in the current review, such as experiences of collective suffering, the impact of TPVs, and the detrimental harm caused by racism and discrimination (Kenny et al., 2022; Theisen-Womersley, 2021; Ziersch et al., 2020). However, the population-focused approach of the current review provided a deeper, more nuanced understanding of how the specific experiences of Hazara refugees shaped their mental health and wellbeing outcomes post-migration. For example, while Alemi et al.’s (2014) review of refugees from Afghanistan highlighted how vulnerability to psychological distress is often rooted in trauma experienced in Afghanistan, the current review was able to directly link individuals’ traumatic encounters in Afghanistan to the enduring ethnic and religious persecution of Hazara people. Without this focused approach, population-specific trauma such as this risks being overlooked or reduced to fit into broader, more general themes developed in conjunction with the experiences of other refugee populations. This discussion underscores an important need for researchers and practitioners to move beyond a one-size-fits-all approach to refugee mental health, and to appropriately engage with the distinct histories, traumas, and strengths of different refugee groups.

## Limitations

This review has some limitations worth noting. First, this review only included Australian articles, potentially limiting the generalisability of findings to Hazara refugees in other host or resettlement countries. Second, the exclusion of non-English publications and gray literature may have resulted in a biased sample. Furthermore, to ensure the inclusion of studies reporting only on the experiences of Hazara refugees, studies were excluded if they did not report participants’ ethnicity. As a result, it is possible that some relevant studies were missing. Third, consistent with a scoping review methodology, this article aimed to provide a comprehensive overview of the available literature regardless of methodological quality (Tricco et al., 2018). Thus, a quality appraisal of included articles was not conducted, and consequently, the methodological rigor of included studies was not assessed, which also introduces a risk of bias. Lastly, through a reflexive lens, we as the authors recognize that who we are and what we bring to the research topic is an integral part of the knowledge we have produced in this review (Braun & Clarke, 2021). Therefore, despite our efforts to recognize any blind spots in our reflexivity, we acknowledge that the discussions presented in this article are inevitably impacted by our subjectivity.

## Conclusion

This review synthesized literature reporting on the mental health and wellbeing of Hazara refugees in Australia, drawing attention to the continuum of the refugee experience, and the cumulative impacts that all stages of forced migration may have on post-migration outcomes. Findings shed light on the collective experiences of suffering and strength within the Hazara community, and emphasize the urgent need to address long-term systemic barriers to wellbeing such as the existing policy restrictions to family reunification. This review also highlights how recognizing the co-occurrence of distress and growth among Hazara refugees is critical for fostering a more in-depth and nuanced understanding of their mental health and wellbeing experiences. Furthermore, the outcomes of the population-specific approach to this review emphasize the benefits of researchers and practitioners prioritizing the distinct histories, traumas, and strengths of different refugee groups, moving beyond a one-size-fits-all approach to understanding refugee mental health and wellbeing post-migration.

## Supplemental Material

sj-docx-1-tva-10.1177_15248380251316905 – Supplemental material for The Mental Health and Wellbeing of Hazara Refugees in Australia: A Scoping Review Supplemental material, sj-docx-1-tva-10.1177_15248380251316905 for The Mental Health and Wellbeing of Hazara Refugees in Australia: A Scoping Review by Grace Sultani, Milena Heinsch, Kate Vincent and Caragh Brosnan in Trauma, Violence, & Abuse
