# Supplementary material for: The Mental Health and Wellbeing of Hazara Refugees in Australia: A Scoping Review
Source: Trauma Violence Abuse. 2025 Feb 13;27(2):461–74. doi: 10.1177/15248380251316905 (PMC12953646; doi:10.1177/15248380251316905)
Supplement: sj-docx-1-tva-10.1177_15248380251316905 – Supplemental material for The Mental Health and Wellbeing of Hazara Refugees in Australia: A Scoping Review [file sj-docx-1-tva-10.1177_15248380251316905.docx]

**Supplementary Table.** Summary of Key Findings Across the Included Articles

| **Author and date** | **Summary of key findings relating to the review** |
| --- | --- |
|  |  |
| **Burford-Rice et al. (2022)** | - Hazara women face multiple social and cultural barriers to help seeking. These barriers include a negative stigma, language and communication difficulties, domestic violence, a preference for informal help-seeking, and cultural differences in the way mental health is conceptualised. |
| **Copolov et al. (2018)** | - Several factors were found to predict personal wellbeing, including acculturation, an absence of trauma symptoms, and having immediate family in Australia. |
| **Copolov & Knowles (2021)** | - Social and emotional support from family, friends, and teachers positively influenced young Hazara refugees’ ability to adapt to the Australian culture. - Post-migration experiences, including challenges and opportunities, differed between Hazara women and men. |
| **Copolov & Knowles (2023** | - Mental health and wellbeing experiences differed between Hazara women and men in Australia, influenced by prevailing gender roles and expectations. - Women relied on religious practices to cope with distress, while some men used substances such as alcohol, smoking, and illicit drugs to minimise feelings of distress. - Participants reported a dissatisfaction with mental health services due to a lack of cultural relevance or usefulness. |
| **Goodall & Hekmat (2021)** | - The memories and experiences of all stages of the refugee journey differed between women and men. - Participants spoke of their ongoing grief post-migration, mourning the loss of their homeland and the natural environment of Afghanistan. - Due to past traumas, feelings of insecurity, anxiety, and distress continued post-migration. |
| **Hamrah et al. (2020)** | - High level depressive symptoms were more common among Hazara women than men. - Depressive symptoms were significantly increased by isolation. |
| **Hamrah et al. (2021)** | - In total, 81.2% of participants recognised that they had a mental health concern. However, only 46.9% of these participants sought professional mental health care. - Family separation was strongly associated with post-traumatic stress disorder. |
| **Iqbal et al. (2012)** | - Participants experienced social difficulties, racism and discrimination, and struggles negotiating the differences between Hazara and Australian culture. Many of these issues were influenced by gender. - The impacts of past trauma and hardship continued to affect participants post-migration. - Participants expressed a strong desire for education and career success in Australia. |
| **Mackenzie & Guntarik (2015)** | - Participants often felt that they held multiple identities post-migration, with many having a strong connection with their Hazara identity. - Participants spoke of feeling determined, and at times responsible, to ‘give back’ to the refugee community. This was often achieved through advocacy and political efforts to support fellow refugees. |
| **Neve (2022)** | - Participants highlighted the negative impacts of Temporary Protection Visas (TPVs) post-migration, including feelings of ongoing uncertainty and insecurity. - Formal and informal supports helped participants cope with psychological distress and resettlement challenges post-migration. - This study reported a determination and drive among Hazara refugees to overcome resettlement barriers and hardship. |
| **Parkes (2020)** | - Hazara refugees relied on various strategies to cope with hardship post-migration and to promote their wellbeing, including social support and sport. For some participants, sport also facilitated a sense of belonging in Australia. - Participants navigated multiple identities during their resettlement. |
| **Phillips (2005)** | - This study highlights the various types of hardship encountered post-migration, including the distress of family separation, prolonged detention, the ongoing impacts of past trauma, and the detrimental effects of TPVs. |
| **Phillips (2011)** | - This paper highlights the ongoing mental health and wellbeing impacts of past trauma among Hazara refugees, and draws attention to the shared, collective memories of persecution experienced by this population. - Participants emphasise the detrimental effects of TPVs, and the distress caused by family separation. - Findings also highlight the co-occurrence of suffering and resilience among this population. |
| **Phillips (2019)** | - The participant interviewed experienced collective suffering, with memories of persecution against the Hazara community having an ongoing impact on their mental health and wellbeing. - TPV contributed to feelings of exclusion and isolation among the participant. - Some members of the Australian community provided social and emotional support, as well as practical assistance such as finding accommodation. |
| **Radford & Hetz (2021)** | - Participants negotiated multiple identities post-migration. - Some participants felt a strong connection to their Hazara identity in Australia, which often resulted in a preference for identifying as ‘Hazara’ rather than ‘Afghan’. - Experiences of racism and discrimination were common for Hazara refugees in Australia. |
| **Russo et al. (2015)** | - Accessing support services was negatively impacted by stigma, and by concerns regarding cultural differences. - Strengthened social support, and religion supported participants’ emotional wellbeing. - Participants expressed a preference for mental health support to come from informal support. |
| **Saberi et al. (2021)** | - Participants described a sense of collective suffering, positioning their own distress within the context of the broader Hazara community. - Past trauma and memories continued to impact the mental health and wellbeing of Hazara refugees post-migration. - Post-migration difficulties included visa restrictions, family separation, and language and cultural differences. - Some barriers to mental health help seeking included stigma, a lack of mental health literacy, language and cultural barriers, and a lack of cultural competency among practitioners. |
| **Saniotis & Sobhanian (2008)** | - Findings highlight the interconnectedness of mind and body among Hazara refugees. - Psychological distress and other mental health and wellbeing concerns were often recognised and expressed as physical symptoms. - In expressing distress, participants often used body-related metaphors. |
| **Shahimi et al. (2023)** | - Participants described their identity as including experiences of both adversity, and resilience. - For some, the pain of pre-migration and transit experiences continued to be felt in their post-migration environment. - Participants’ ethnic identity and community history positively influenced their sense of resilience and their personal sense of identity. - Participants’ Hazara identity influenced educational and career goals, often focused on advocacy and activism efforts for their community. - At some point, almost all participants struggled to feel a sense of belonging in Australia, due to racism, social exclusion, and immigration policies. - Participants spoke of the benefits of socially connecting with others who have experienced similar trauma and hardship, particularly fellow Hazaras. |
| **Spaaij et al. (2023)** | - Playing the traditional sport of Sangarag fostered a feeling of community and belonging among Hazara refugees in Australia. Participating in the sport helped participants expand their social networks, and provided a safe space for participants to share their distress or struggles. - Participants spoke of encountering racism and discrimination while playing Sangarag. |
| **Wilson et al. (2023)** | - Findings highlight gendered mental health and wellbeing experiences in Australia as influenced by traditional gender roles and expectations. |
